# Effect of Single-Dose and Short-Term Administration of Si Jun Zi Tang on the Pharmacokinetics of Gefitinib in Rats

**DOI:** 10.1155/2021/6655449

**Published:** 2021-07-26

**Authors:** Ying Li, Xiaowei Zhou, Ming Niu, Mingyu zhang, Qiong Gu, Wei Chen, Bin Dong, Yuanyuan Zhang, Ruisheng Li, Chunyu Li, Guohui Li

**Affiliations:** ^1^Department of Pharmacy, National Cancer Center/National Clinical Research Center for Cancer/Cancer Hospital, Chinese Academy of Medical Sciences and Peking Union Medical College, Beijing, China; ^2^Integrative Medical Center, The Fifth Medical Center of Chinese People's Liberation Army General Hospital, Beijing, China; ^3^Research Center for Clinical and Translational Medicine, The Fifth Medical Center of Chinese People's Liberation Army General Hospital, Beijing, China

## Abstract

**Background:**

Si Jun Zi Tang (SJZ), a four-herb Chinese medicine formula that has been described for approximately one thousand years, is often prescribed for cancer patients as a complementary therapy in China. However, the mechanism by which Si Jun Zi Tang enhances the efficacy of gefitinib is unclear.

**Methods:**

We investigated how Si Jun Zi Tang affected the pharmacokinetics of gefitinib in rats. A rapid, specific, and reliable ultra-performance liquid chromatography method with mass spectrometry was established to determine the plasma concentration of gefitinib.

**Results:**

The results showed that a single intragastrically administered dose of Si Jun Zi Tang increased the pharmacokinetic parameters of gefitinib (*C*_max_, 3156.13 *μ*g/L; A UC, 46281.5 *μ*g/L/h) by 3 folds in rats compared with the administration of gefitinib alone (*C*_max_, 1352.07 *μ*g/L; AUC, 11823.7 *μ*g/L/h). Si Jun Zi Tang could also alter the pharmacokinetics of gefitinib by prolonging the time to reach *C*_max_.

**Conclusions:**

Potential pharmacokinetic interactions between gefitinib and SJZ were evaluated, and SJZ extended *T*_max_ and T1/2 and increased the *C*_max_ and AUC of gefitinib. Long-term administration of gefitinib in combination with Si Jun Zi Tang would improve the efficacy of gefitinib.

## 1. Introduction

Gefitinib is a selective small-molecule epidermal growth factor receptor (EGFR) tyrosine kinase inhibitor (TKI) that is approved for the treatment of advanced non-small-cell lung cancer (NSCLC) with mutations of EGFR that are sensitive to gefitinib [1, 2]. Gefitinib is metabolized in the liver by cytochrome P450 (CYP) enzymes, mainly CYP3A4 [3]. Thus, it is susceptible to drug-drug interactions with any coadministered drug, and it has been known to be an inhibitor or inducer of CYP450 enzymes [3–5]. It is necessary to monitor the clinical application of gefitinib to ensure efficacy and to prevent adverse reactions.

Si Jun Zi Tang (SJZ) was first described in the “Prescription People's Welfare Pharmacy” approximately one thousand years ago for the treatment of gastrointestinal disorders, and it can effectively treat nausea, vomiting, and diarrhoea [6]. SJZ is a traditional Chinese medicine compound, and its main ingredients are four traditional herbal medicines, namely, *Panax ginseng*, *Atractylodes macrocephala Koidz*, *Poria cocos*, and *Nardostachys jatamansi*. Recently, the use of herbal medicines in combination with antitumour drugs has developed considerable interest [7, 8]. It plays a critical role in improving the quality of life of chemotherapy patients with malignant tumours such as lung cancer, colon cancer, and gastric cancer [9, 10]. It has been found that SJZ indeed improved the quality of life of chemotherapy patients, and it was used in the clinic in combination with chemotherapeutics for the treatment of cancer [10]. Emerging evidence has shown that SJZ can play a significant role in suppressing tumours and has a protective effect against gastrointestinal mucosa damage induced by chemotherapy [9]. Some studies [11–16] have shown that Chinese herbal ingredients in Si Jun Zi decoction influence the CYP450 enzyme. The Liang Yang group [11] found that ginseng reduced the metabolic rate of midazolam and caffeine, which were metabolized by CYP450 enzymes.

A previous study showed that SJZ combined with gefitinib could inhibit tumour growth and cancer cell metastasis to the lungs in a mouse model of Lewis lung cancer cells [17]. Nevertheless, the mechanism by which SJZ enhances the efficacy of gefitinib is unclear. We established an ultra-performance liquid chromatography method with mass spectrometry detection (UPLC-MS/MS) to determine the gefitinib concentration in rat plasma and studied the effect of SJZ on the pharmacokinetics (PK) of gefitinib.

## 2. Methods

### 2.1. Materials and Reagents

Gefitinib was purchased from Macklin Biochemical Co., Ltd. (Shanghai, China). The reference standards of gefitinib (purity 99.8%) and propranolol hydrochloride (purity 100%, internal standard) were obtained from the National Institutes for Food and Drug Control (Beijing, China). Si Jun Zi Tang consisting of *Panax ginseng* C. A. Mey. (roots), *Atractylodes macrocephala* Koidz. (roots), *Poria cocos* (Schw.), Wolf (sclerotia), and *Nardostachys jatamansi* DC were provided by Beijing Lüye Pharmaceutical Company.

HPLC-grade solvents, including methanol and acetonitrile (Thermo Fisher, MA, USA), were used in the study. HPLC-grade formic acid was purchased from Thermo Fisher (MA, USA), and the water was Wahaha purified (Shenyang, China).

### 2.2. Instrumentation

UPLC-MS/MS analysis was carried out on a Waters Model Xevo TQD separation system (Waters, Milford, MA, USA) supplied with a sample manager and a binary solvent manager. The instrument was also equipped with electrospray ionization mass spectrometry in multiple reaction monitoring (MRM) mode using a triple-quadrupole mass spectrometric detector (Waters, Milford, MA, USA). The data were acquired and processed using the MassLynx TM Version 4.1 software (Micromass, Manchester, UK).

### 2.3. Preparation of Calibration Standards and QC Samples

Stock solutions of gefitinib were prepared from two independent weightings, one for the calibration standards and the other for the quality control (QC) samples. Stock solutions (1.0 mg/mL) were prepared by dissolving gefitinib in methanol. Propranolol was used as an internal standard (IS) and was dissolved in methanol to obtain a 1.0 *μ*g/mL working solution. All solutions were kept at −20°C and were increased to room temperature before use.

The stock of gefitinib at 1.0 mg/mL was diluted with methanol to obtain a standard solution containing 10 *μ*g/mL of gefitinib, and then the standard sample was diluted with rat blank plasma to achieve final concentrations of 2000, 1500, 1000, 500, 200, 50, 20, and 10 *μ*g/L. Using the same method, QC samples with high, medium, and low concentrations were prepared with blank plasma at 1400, 300, and 30 *μ*g/L.

### 2.4. Sample Preparation

A simple protein precipitation method was carried out to extract gefitinib from all plasma samples. The IS was diluted with acetonitrile to a final concentration of 100 *μ*g/L. IS (450 *μ*L) was pipetted into 150 *μ*L of plasma, vortexed for 2 min, and centrifuged at 13000 rpm for 10 min to precipitate the protein. The supernatant was collected and evaporated to a dried residue by a speed vacuum concentrator. The dried residue was reconstituted with 100 *μ*L of the initial mobile phase and centrifuged at 13000 rpm for 5 min. Portions of the 10 *μ*L volume were directly injected into the UPLC-MS/MS system.

### 2.5. Preparation of the Test Substance

The powder obtained from gefitinib was dissolved in 0.5% sodium carboxymethyl cellulose (CMC-Na) at a concentration of 4.5 mg/mL. SJZ comprises four commonly used herbs, *Panax ginseng* C. A. Mey. (roots), *Atractylodes macrocephala Koidz*. (roots), *Poria cocos* (Schw.), Wolf (sclerotia), and *Nardostachys jatamansi* DC. (roots), in the ratio of 9 : 9 : 9 : 6, respectively. The quality of each herb conformed to the requirements of the standards specified by the Chinese Pharmacopoeia [18]. The dried aqueous extract of SJZ was prepared by twice refluxing the four herb powders for 30 min with 8 volumes (vs. four herb weights) of water, and then the water extracts were pooled and concentrated under reduced pressure and finally dried to a powder under a low temperature.

### 2.6. Pharmacokinetic Experiments in Rats

Male Sprague-Dawley rats weighing 280 ± 30 g were supplied by Beijing Vital River Laboratory Animal Technology Co., Ltd. (Licence No. SYXK 2014-0003) (Beijing, China). The animals were kept in a room at 22–24°C with 55–60% relative humidity and a light cycle (12-hour light and 12-hour dark). They had free access to standard rodent chow and clean water ad libitum. The rats were fasted for 12 hours before the experiments while water was freely supplied.

The healthy rats were divided into six groups of six rats each. The control group was treated orally with gefitinib (45 mg/kg) that was dissolved in 0.5% CMC-Na. SJZ at an equivalent dose (2.97 g/kg), three times the equivalent dose (8.91 g/kg), six times the equivalent dose (17.82 g/kg), and twelve times the equivalent dose (35.64 g/kg) was administered by gavage to rats in groups A-D, and 1 h later, the rats in groups A-D were given gefitinib at a dose of 45 mg/kg.

**Figure 1:**
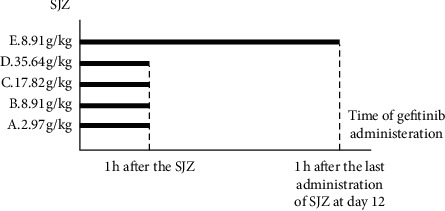
The experimental group and the dosing scheme.

SJZ (8.91 g/kg) was administered by gavage to six rats daily for 12 consecutive days in group *E* to examine the effects of multiple doses of the herbal medicines one hour after the last administration of SJZ at day 12, and gefitinib (45 mg/kg) was administered (Figure 1).

Blood samples (0.5 mL) were collected from the fosse orbital veins and were placed into heparinized polythene tubes at 0.25, 0.5, 1, 1.5, 2, 3, 4, 6, 8, 12, and 24 h after dose. The supernatant of blood samples was collected after centrifugation at 4000 rpm for 10 min and stored at −80°C until analysis. The pharmacokinetics analysis was performed by a noncompartmental approach using DAS version 2.0 (China).

### 2.7. Statistical Analysis

Statistical analyses were performed using SPSS software (SPSS for Windows, version 22.0, IBM Corp, Armonk, NY, USA). Noncompartmental PK parameters are presented as the mean ± standard deviation (mean ± SD) and were analysed with unpaired *t*-tests for comparisons between two groups or a single-factor ANOVA followed by the Dunnett tests for comparisons among multiple groups. *P* values less than 0.05 were considered statistically significant.

## 3. Results

### 3.1. Mass Method Development

**Figure 2:**
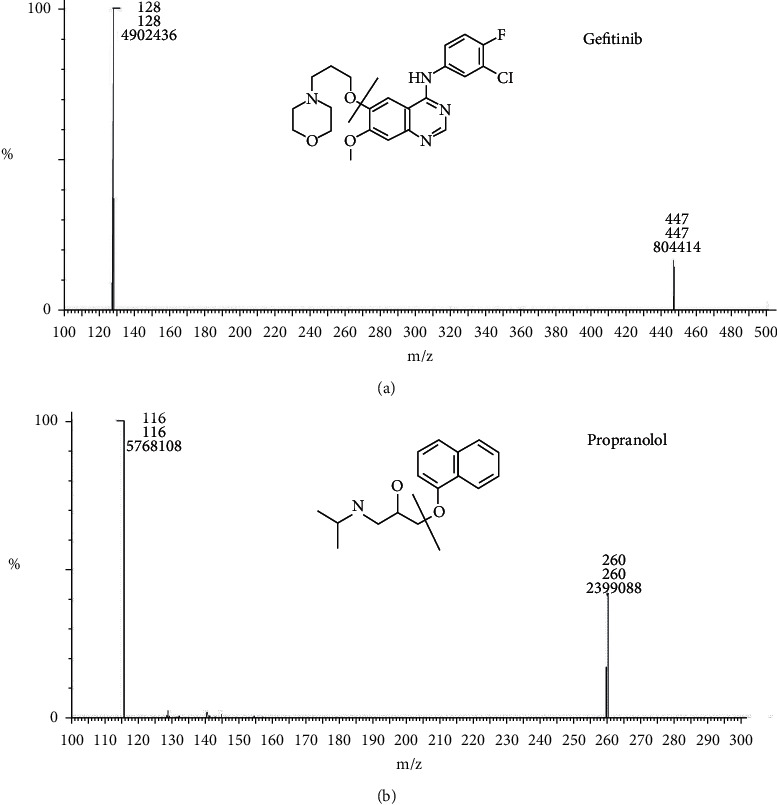
The chemical structures and MS/MS spectra of gefitinib (a) and propranolol (b).

The mass spectrometric parameters were optimized with the corresponding standard solutions. The final analysis was conducted in positive ionization mode since it gave a good response to gefitinib and IS, which was consistent with previous reports [1, 5, 12]. In the precursor ion full scan spectra, [M + H]+ fragments were the most abundant at m/z 447.3 (gefitinib) and m/z 260.0 (IS), and the products selected for quantification were at m/z 127.8 (gefitinib) and m/z 116.1 (IS) (Figure 2). MS/MS parameters, including collision energy, cone voltage, capillary voltage, desolvation temperature, ESI source temperature, collision gas flow rate, cone gas flow, and desolvation gas flow rate, were optimized to obtain the highest response of the protonated molecules of the studied compounds.

### 3.2. Optimization of Chromatographic Conditions

A mixture of gefitinib and IS was eluted using different mobile phases composed of mixtures of methanol, water, and formic acid. The percentage of acetonitrile in the mobile phase had a significant effect on the shape and retention time. Gradient elution was used to ensure the preferable peak shape and retention time of the two compounds. Moreover, formic acid was essential to obtain sharp peaks for gefitinib. The final analysis was thus performed with gradient elution using a mobile phase of acetonitrile and water with 0.1% (v/v) formic acid. The shortest analytical run time of the present method was 5 min, although all compounds were eluted sufficiently within 2.5 min after injection. The remaining 2.5 min was spent diminishing the memory effect from the column and stabilizing it before the next injection.

### 3.3. Method Validation

#### 3.3.1. Specificity

**Figure 3:**
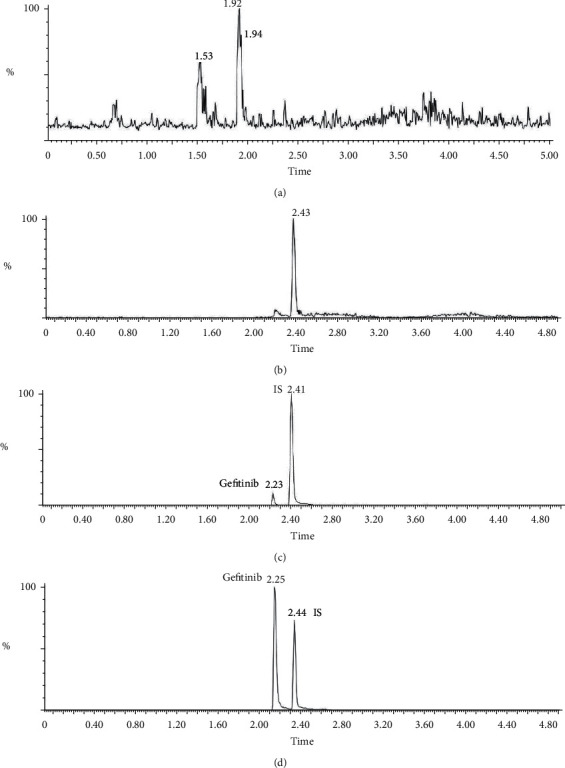
Representative chromatograms of blank plasma (a), blank plasma spiked with 10 *μ*g/L IS (b), blank plasma spiked with 10 *μ*g/L gefitinib (the lowest limit of quantification) and 100 *μ*g/L IS (c), and rat plasma sample 1 h after a single oral dose of 45 mg/kg gefitinib (d).

The specificity was thoroughly assessed by screening the chromatograms of rat blank plasma spiked with gefitinib and propranolol (Figure 3). No endogenous interference was observed at the retention time of gefitinib (2.23 min) and IS (2.43 min). This result indicated that the developed method had efficient specificity under the working conditions.

#### 3.3.2. Linearity of the Calibration Curves and the Limit of Quantification

Calibration curves were fitted by a linear weighted (1/*x*2) least squares regression method over the concentration range of 10–2000 *μ*g/L for gefitinib. The linear equation was *y* = 0.001*x* − 0.014 with a correlation coefficient *r* > 0.9995. The deviation from the nominal concentration should be within ±20% for the lowest limit of quantification (LLOQ). Based on the criteria, the LLOQ value was set at 10 *μ*g/L, and the measured results are presented in Table 1. Carryover was tested by injecting two processed blank matrix samples sequentially after injecting an upper limit of quantification (ULOQ) sample. Carryover should not exceed 20% of the LLOQ in the blank samples. ULOQ was set at 2000 *μ*g/L, and to ensure the analysis of samples over ULOQ, dilution integrity was carried out by diluting an ultrahigh QC sample at ten times the highest calibration level.

#### 3.3.3. Accuracy and Precision

The mean concentration values for the QC samples at 30, 300, and 1400 *μ*g/L were estimated using six replicates per day and run repeated on three separate days to determine the intra-/interday accuracy and precision. The intraday and interday precisions of gefitinib were within ±15% of the nominal concentration (Table 1).

#### 3.3.4. Recovery and Matrix Effect

Recovery was assessed by spiking plasma samples with gefitinib at 30, 300, and 1400 *μ*g/L, along with propranolol (IS). Plasma samples were then processed, and the drugs were extracted as described in the experimental section. The extraction recovery was determined in sextuplicate by comparing the three concentrations of samples with reference solutions containing blank plasma extracts spiked with gefitinib at the same concentrations. The recoveries are presented in Table 2. Recovery values of not less than 86.64 indicated good extraction efficiency of the proposed sample treatment method. The matrix effect was assessed in sextuplicate by comparing the concentrations obtained with the three solutions at 30, 300, and 1400 *μ*g/L in blank plasma extracts with the same solutions in acetonitrile for gefitinib. The matrix factor of not more than 9.52 (Table 2) showed that a negligible matrix effect was found for plasma.

#### 3.3.5. Stability Study

Gefitinib stability in rat plasma was assessed at three concentrations (30, 300, and 1400 *μ*g/L) in sextuplicate. The processed samples were left in the autosampler (10°C) for 48 h to study the autosampler stability. Short-term stability was evaluated by detecting the samples left at room temperature (25°C) for 12 h. The samples were stored at −20°C for 60 days for the assessment of long-term stability. Three freeze (at approximately −20°C)-thaw (room temperature) cycles were used to assess the freeze-thaw stability. The results of the stability testing are reported in Table 3 and indicated the stability of gefitinib under the abovementioned conditions since the recoveries were within the acceptance criteria (±15%). In addition, plasma samples exceeding the upper limit of the assay were adequately diluted 10 times with blank plasma in this study, and the recovery of samples diluted 10 times was 102.43% (RSD = 4.41%).

### 3.4. Pharmacokinetics Studies in Rats

The parameters of gefitinib are reported in Table 4. In the control group, gefitinib was rapidly absorbed within 3 h of administration, and the elimination half-life (T1/2) was 4.79 h. A longer time to reach the maximum concentration (*T*_max_), T1/2, and the area under the curves (AUC) were observed in the rats that were pretreated with SJZ prior to the gefitinib dose, and a higher maximum concentration (*C*_max_) was reported. In addition, clearance (CL_z_/F) was lower than that in the control group.

#### 3.4.1. Effect of SJZ Dosage on Gefitinib and PK

**Figure 4:**
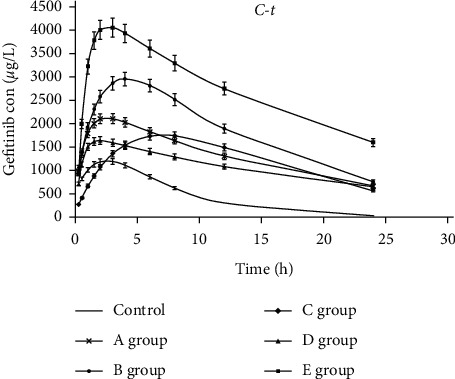
Average plasma concentration-time profiles of gefitinib in the control group and in rats pretreated with SJZ 2.97 g/kg (A group), 8.91 g/kg (B group), 17.82 g/kg (C group), 35.64 g/kg (D group) 1 h or 8.91 g/kg SJZ once daily for 12 days (E group) prior to gefitinib administration.

The average concentration-time profiles of gefitinib in the rats receiving different doses of SJZ are shown in Figure 4. There were prolonged *T*_max_ and T1/2 and a higher *C*_max_ and AUC in these groups. According to this study, the *C*_max_ (3156.13 *μ*g/L) was significantly increased only in the group with three times the equivalent dose (8.91 g/kg) of SJZ (*P*=0.007), as well as the group with six times the equivalent dose (17.82 g/kg) of SJZ (*C*_max_: 2888.32 *μ*g/L, *P*=0.028) compared with the control group. *T*_max_ was prolonged to 4.5 h, and the average AUC_0-t_ was 46281.5 *μ*g/L/h in the group treated with three times the equivalent dose of SJZ. The CL_z_/F of gefitinib in all groups treated with SJZ was significantly lower than that of the control (*P* < 0.05). Three times the equivalent dose of SJZ had the greatest influence on the PK of gefitinib. *C*_max_ and AUC_0-t_ of gefitinib increased with increasing SJZ dose in the range of 2.97 g/kg–8.91 g/kg, while *C*_max_ and AUC_0-t_ decreased when the SJZ dose exceeded 8.91 g/kg.

#### 3.4.2. Effect of Multiple Doses of SJZ on Gefitinib and PK

The concentration-time profiles in Figure 4 show that the PK data of gefitinib after the rats were pretreated with SJZ (8.91 g/kg) once daily for 12 consecutive days was similar to the single dose (8.91 g/kg), while *C*_max_ (4934.85 *μ*g/L) and AUC_0-t_ (73049.4 *μ*g/L/h) were higher than the rats with the single-dose and the difference was statistically significant (*C*_max_: *P* = 0.029; AUC_0-t_: *P*=0.011). The CL_z_/F (0.39 L/h/kg) was lower than that of the group with a single dose. A long-term administration of gefitinib in combination with SJZ increased the concentration of gefitinib in the rats.

## 4. Discussion

The UPLC-MS/MS method is becoming an important tool for clinical laboratories because it is a very specific, selective, and sensitive technique. Additionally, this permits a wide range of applications, and it is possible to obtain a large number of quantitative or qualitative results. In this study, we successfully established a UPLC-MS/MS method to determine the concentration of gefitinib. We used a protein precipitation method to prepare samples rather than a solid-phase extraction [5]. This method was simple and convenient to fully precipitate the protein. The accuracy, precision, recovery, and matrix effect in the study were within ±15% of the nominal concentration, which complies with the FDA standards. Diverse studies have shown gefitinib stability in plasma at room temperature, +10°C, and −20°C. The Nan Zheng group [1] tested gefitinib at −80°C for 30 days, and the Chi-Kyoung group [19] verified that gefitinib was stable at −20°C and −70°C for 2 weeks. Our study showed that gefitinib was stable at −20°C for at least eight months, with good accuracy and precision. The LLOQ value was set at 10 *μ*g/L, but it was sufficient for the detection of gefitinib in rat plasma. The study was conducted using plasma with a wide range from 10 to 2000 *μ*g/L, and plasma samples exceeding the upper limit of the assay were adequately diluted 10 times with blank plasma. The stability of dilution was considered acceptable.

The parameters of the noncompartmental model showed that *C*_max_ increased by 75.8% in the group that was given gefitinib 1 h after equivalent-dose SJZ treatment, and the AUC increased by 175.9%. In addition, *T*_max_ was prolonged to 3.17 h, and T1/2 was prolonged to 13.0 h. The CLz/F was lower in the groups that received SJZ than in the control group, and the difference was statistically significant. It was deduced that SJZ could alter the PK process by slowing gefitinib clearance. In parallel, the absorption rate, which was reflected by *T*_max_, was also significantly delayed by SJZ coadministration. Long-term administration of gefitinib in combination with SJZ increased the concentration of gefitinib in patients and increased drug exposure. It is worth noting that *C*_max_ and AUC_0-t_ of gefitinib increased with increasing SJZ dose in the range of 2.97 g/kg–8.91 g/kg, while *C*_max_ and AUC_0-t_ decreased when the SJZ dose exceeded 8.91 g/kg. The volumes of SJZ were 4.4 ml and 3.5 ml for doses of 17.82 g/kg and 35.64 g/kg, respectively. The results may be due to the large volume of gavage, which affects absorption, and it is necessary to carry out more research in the pharmacodynamics of SJZ to determine the optimal dose.

The changes in the pharmacokinetic characteristics of gefitinib may be related to the effect of Si Jun Zi decoction on CYP450 enzymes. Gefitinib is metabolized in the liver by CYP3A4 [3]. The concurrent use of other drugs and gefitinib may augment or oppose the pharmacokinetics or pharmacodynamics, which can then increase or decrease the pharmacological or toxicological effects of either constituent. Some studies have shown that Chinese herbal ingredients in Si Jun Zi decoction influence CYP450 enzymes. A study [11] showed that ginseng reduced the CL of midazolam and caffeine, which were metabolized by CYP450 enzymes. CYP3A2 (an isozyme of CYP3A4) and gene expression levels in Sprague-Dawley rats exposed to *P. ginseng* extracts were evaluated by western blotting and quantitative PCR in the study [11] and compared with the control groups, *P. ginseng* downregulated the expression of CYP3A2 at both the protein and gene levels. Another study [12] indicated that repeated administration of Sailuotong induced CYP1A2 and CYP2C11 but significantly inhibited CYP3A1/3A2 in rats, and these impacts were attributed to the ingredients of ginseng and gingko individually or cooperatively to a large extent. Several in vitro studies [13–15] asserted that ginsenosides Rb1, Rb2, Rc, Re, and Rg1 did not affect CYP enzyme activity but that their metabolite ginsenoside Rh2 did inhibit CYP1A2, CYP2C9, and CYP3A4. In addition, bioactive components of *Glycyrrhiza uralensis* inhibited the expression of CYP2D6, CYP2E1, and CYP3A4 [16]. Ginseng and *Glycyrrhiza* are components of SJZ, so we speculate that certain components of SJZ can inhibit CYP450 enzymes, thereby slowing the rate of gefitinib metabolism and increasing exposure. More studies on basic pharmacology are needed. Currently, it is essential to carry out therapeutic drug monitoring for patients treated with combinations of SJZ and gefitinib.

## 5. Conclusions

A simple, rapid, and sensitive UPLC-MS/MS bioanalytical method has been developed and validated for the quantification of gefitinib in rat plasma. The method was successfully applied to PK determination following the oral administration of two combinations of gefitinib and SJZ to rats. Potential pharmacokinetic interactions between gefitinib and SJZ were evaluated, and SJZ prolonged *T*_max_ and T1/2 and increased *C*_max_ and AUC of gefitinib. Thus, SJZ may enhance the efficacy of gefitinib by changing the pharmacokinetics of gefitinib. These findings should be noted in clinical therapeutics, especially when using two concurrent drugs which may have drug-drug interactions.

## Figures and Tables

**Table 1:** Summary of the accuracy and precision of gefitinib analyses in rat plasma.

Nominal con. (*μ*g/L)	Intraday			Interday		
	Measured con. (*μ*g/L) (mean ± SD)	Accuracy (%)	Precision (%)	Measured con. (*μ*g/L) (mean ± SD)	Accuracy (%)	Precision (%)
10 (LLOQ)	11.32 ± 0.41	113.05	3.47	10.57 ± 1.15	105.74	10.81
30	32.12 ± 1.82	107.05	5.66	31.40 ± 2.35	104.69	7.48
300	319.62 ± 11.51	106.53	3.59	313.49 ± 22.04	104.49	7.04
1400	1457.25 ± 55.86	104.08	3.86	1429.16 ± 105.91	102.08	7.41

**Table 2:** Matrix effect and extraction recovery of gefitinib.

Nominal con. (*μ*g/L)	Recovery		Matrix effect	
	Mean ± SD (%)	RSD (%)	Mean ± SD (%)	RSD (%)
30	107.14 ± 7.43	6.94	100.62 ± 4.93	4.90
300	97.78 ± 7.64	7.82	112.70 ± 9.52	8.44
1400	86.64 ± 2.96	3.42	102.80 ± 4.00	3.89

**Table 3:** Evaluation of the stability of gefitinib in rat plasma.

Condition	Nominal con. (*μ*g/L)	Mean recovery ± SD (%)	RSD (%)
Autosampler stability	30	87.95 ± 2.06	2.34
	300	87.05 ± 1.53	1.76
	1400	87.93 ± 1.66	1.89

Short-term stability	30	88.23 ± 1.42	1.61
	300	88.92 ± 2.31	2.60
	1400	86.60 ± 1.20	1.39

Long-term stability	30	91.50 ± 2.17	2.36
	300	90.28 ± 2.95	3.26
	1400	90.40 ± 2.93	3.24

Freeze-thaw stability	30	87.23 ± 1.88	2.15
	300	89.37 ± 3.67	4.11
	1400	88.12 ± 2.54	2.89

**Table 4:** Noncompartmental PK parameters of gefitinib in rats pretreated with SJZ (mean ± SD).

Parameters	Control group	A group	B group	C group	D group	E group
SJZ dosage	—	2.97 g/kg	8.91 g/kg	17.82 g/kg	35.64 g/kg	8.91 g/kg for 12 days
Interval time	—	1 h	1 h	1 h	1 h	1 h
AUC0-t (*μ*g/L/h)	11823.7 ± 2189.3	32623.6 ± 11490.8^*∗*^	46281.5 ± 11680.1^*∗*^	39454.6 ± 13977.0^*∗*^	27330.9 ± 9689.3	73049.4 ± 16799.9^*∗*†^
AUC∞ (*μ*g/L/h)	12650.0 ± 2551.7	51943.1 ± 41963.3	58475.3 ± 14589.1	101473.5 ± 73686.7^*∗*^	50095.0 ± 28295.6	131044.8 ± 55149.9^*∗*†^
MRTt (h)	6.45 ± 1.3	8.91 ± 0.7^*∗*^	9.42 ± 0.8^*∗*^	10.38 ± 0.8^*∗*^	9.74 ± 1.2^*∗*^	10.00 ± 0.7
MRT∞ (h)	7.04 ± 1.5	14.68 ± 4.6	15.99 ± 5.8	8.17 ± 4.2	20.64 ± 11.1^*∗*^	24.44 ± 12.4^*∗*^
*C*_max_ (*μ*g/L)	1352.07 ± 247.1^*∗*^	2377.48 ± 536.9	3156.13 ± 993.2^*∗*^	2888.32 ± 982.3	1899.28 ± 785.4	4934.85 ± 1356.3^*∗*†^
*T*_max_ (h)	2.08 ± 0.8	3.17 ± 2.5	4.50 ± 2.2	10.67 ± 2.1^*∗*^	3.93 ± 3.9	4.92 ± 4.2
T1/2z (h)	4.79 ± 2.0	13.00 ± 9.9	10.74 ± 4.4	6.72 ± 2.7	17.22 ± 9.2	21.11 ± 18.1^*∗*^
CL_z_/F (L/h/kg)	3.67 ± 0.7^*∗*^	1.26 ± 0.7^*∗*^	0.81 ± 0.2^*∗*^	0.78 ± 0.6^*∗*^	1.15 ± 0.6^*∗*^	0.39 ± 0.2^*∗*†^
V_z_/F (L/kg)	24.06 ± 6.8^*∗*^	17.12 ± 3.5	12.24 ± 4.4	7.67 ± 7.6	24.33 ± 10.9	9.37 ± 3.9^*∗*^

Notes: ^*∗*^*p* < 0.05 versus control group; ^†^*P* < 0.05 versus *D* group. AUC_0-t_: The area under the concentration-time curve from time 0 to the last time point selected; AUC_∞_: the area under the concentration-time curve from 0 to infinity; MRT_t_: the average dwell time from time 0 to the last time point selected; MRT_∞_: the average dwell time from 0 to infinity; *C*_max_: maximum plasma concentration; *T*_max_: the time to reach the maximum plasma concentration; CL_z_/F: clearance rate; V_z_/F: apparent volume of distribution.

## Data Availability

The data used in the current study can be accessed by request via the corresponding author.
